# Thc Alleged Serum Trust

**Published:** 1904-02

**Authors:** 


### Correspondence.

### The Alleged Serum Trust.

*Editor Texas Medical Journal.*

It has been charged repeatedly by sensational newspapers within the past few days that the antitoxin producers of this country have formed a trust and greatly advanced the prices of diphtheria antitoxin without any regard whatever for the welfare of the public. As it is important for the medical profession to know the exact truth, we ask you to allow us space to present the facts to your readers.

The manufacturers of antitoxin have not formed a trust or combination.

The price of antitoxin has not been advanced, but it has been materially reduced.

Formerly diphtheritic antitoxin was marketed in two grades called by us "special" which was a concentrated serum, and "regular," a much weaker product. For obvious reasons, the majority of the medical profession preferred the "special" serum, its much smaller bulk per 1000 units more than offsetting, in their opinion, the necessarily greater cost.

There has also been considerable complaint from the medical profession about the multiplicity of packages and sizes offered, we ourselves listing ten, and it was believed that a reduction of this number would be in the interest of everyone concerned.

For these reasons, and others of equal weight that we could enumerate, we determined to discontinue the weaker serum and supply only the concentrated, or highest grade product, after January 1, 1904 At the same time we discontinued the 500 unit so-called immunizing dose, since many able members of the medical profession now believe that 1000 units should be given for immunizing purposes. At the same time we reduced the price of the concentrated serum as is shown by comparing the present retail price of this product with that of last year.

| | 1000 Units. | 2000 Units. | 3000 Units. | 4000 Units. |
|---|---|---|---|---|
| 1903, Price.................. | $ 2 25 | $ 4 00 | $ 5 75 | Not offered. |
| 1904, Price.................. | 2 00 | 3 50 | 5 00 | $ 6 50 |
| | 25 | 50 | 75 | |

Of course, there are liberal discounts to druggists from these prices.

The charge that we have raised the price of antitoxin is therefore seen to be absolutely false. Not only have we reduced the price, but we are supplying now only the highest grade serum that can be produced by expensive equipment and the most advanced scientific methods, believing that the interests of the public and the medical profession are alike conserved by our doing so. Our house introduced the syringe package of serum to the physicians of America and we have ever striven to co-operate with the medical profession as far as possible. It is, therefore, we believe sufficient to call attention to the above facts, which completely refute the absurd and maliciously circulated charges which have called forth this statement. Very truly yours,

FREDERICK STEARNS & CO.

Detroit, Mich., January 13, 1904.

---

LETTER FROM PARKE, DAVIS & CO.

103 Dearborn Ave., CHICAGO, January 15, 1904.

*Messrs. Parke, Davis & Co., Detroit, Mich.*

GENTLEMEN: There has been a good deal of comment in the newspapers in reference to the alleged combination of the antitoxin manufacturers, with a consequent raise in price, which it is claimed is 50 per cent. over former prices. How much truth is there in this statement? Kindly let me have your side, as we have been requested to take up the matter. Also, will you kindly send me your price list in force three months ago, or more, and that of today, both wholesale and retail. Thanking you in advance for the favor of a reply, I am, respectfully yours,

GEORGE A. SIMMONS, Editor.

*George H. Simmons, M. D., Editor of the Journal of the American Medical Association, 103 Dearborn Ave., Chicago, Ill.*

DEAR SIR: In answer to your inquiry of the 15th, we have pleasure in making the following statement:

Beginning January 1, 1904, we market only one grade of antitoxin, in four packages or doses:

| Number of Units. | List Price. |
|---|---|
| 1000 | $2.00 |
| 2000 | 3.50 |
| 3000 | 5.00 |
| 4000 | 6.50 |

Trade discounts: 25 per cent. to the retail and 33 1-3 per cent. to the wholesale druggist.

*Injecting Device.*—With each package of the "new serum" we now supply an expensive device for administering the contents, sparing the physician the need of buying and sterilizing a serum syringe.

*Last Year's Prices.*—Last year we marketed two grades of antidiphtheritic serum—the standard or X serum, testing 200 and more units to the c. c.; the more powerful and concentrated special or XX testing 500 and more units to the c. c.—at the following prices:

| Number Units. | X Prices. | XX Prices. |
|---|---|---|
| 1000 | $ 1 50 | $ 2 25 |
| 2000 | 3 00 | 4 00 |
| 3000 | 4 50 | 5 75 |
| 4000 | Not formerly listed. | Not formerly listed. |

*Last Year's Discounts.*—Only 20 per cent. to the retailer; to the wholesaler, 33 1-3 per cent.

*Injecting Device.*—None furnished until the close of the year.

*Potency of the New Serum.*—In the 4000 unit package we are placing serum testing on an average 600 units to the c. c.; in the 3000 unit package, serum testing on an average 500 units to the c. c.; in the 2000 unit package, serum testing on an average 400 units to the c. c.; and in the 1000 unit package, serum testing on an average 300 units to the c. c.

Thus, comparing prices and discounts, old and new, allowing for the additional cost of the injecting device, and remembering that the new serum in point of concentration and antitoxic potency, is much superior to the X and all but equal to the XX, you will see that our 1904 prices exhibit a reduction and not an advance.

*Free Exchanges.*—Fully 40 per cent. of all the serum we sell comes back for free exchange. What becomes of the returned serum? It is poured into the sewer. This is the sole reason for our marketing henceforth one grade of antitoxin in place of two, and four packages or doses in place of ten. The producer, the retailer and the wholesaler will be spared a useless trouble and an enormous expense, while the physician loses nothing thereby.

*The Chicago Department of Health.*—For years the department profited by the illegal and unconstitutional action of the New York Board of Health, which sold to Chicago its surplus serum. When Mayor Low and Dr. Lederle discontinued such sale the Department, by playing off one producer against another, succeeded in screwing down the price until its business was altogether unprofitable. We know that every sale of serum ever made the Chicago Department by a private producer has been made at a loss. We see no reason why, in buying antitoxin for its poor, the great city of Chicago should compel us to furnish our product at a loss. How much money will Chicago have to spend for antitoxin to be donated to the poor? Less than $5000 a year. If a single life is sacrificed among the indigent sick of your city, it will be solely because the Department refuses to pay a fair and reasonable price for a good product. As for the diphtheria sufferers who are not charity patients, they will henceforth pay less and not more, quality considered.

*Federal Supervision.*—A federal law compels all makers of biological products to work under federal license and supervision, the license being revocable for faulty equipment of men or appliances, or unsatisfactory product.

Considering the harassing dangers and responsibilities of our biological work, our immense investment of capital, our employment of the most expert scientific talent, and the heavy waste in the form of free exchanges, we feel wholly warranted in writing and urging you to send to Detroit at our expense a trusty and accomplished biologist. Let him come, see our laboratories, compute our expenses and producing costs, and publish his report in your pages. We are prepared to stand or fall by the verdict of any unbiased expert or commission. Very truly yours,

PARKE, DAVIS & CO.

P. S.—Under separate cover we are sending you a copy of our last year's price list (see page 123 and the price change sheet there inserted), and we enclose herewith copy of our latest antitoxin announcement.

---

A QUESTION IN BUGOLOGY.—Question—If germs germinate in Germany and parasites reside in Paris, what will we find in Cork? Answer—Mike-robes.

